# Novel *Ehrlichia canis* genogroup in dogs with canine ehrlichiosis in Cuba

**DOI:** 10.1186/s13071-022-05426-0

**Published:** 2022-08-23

**Authors:** Maylin González Navarrete, Adnan Hodžić, Belkis Corona-González, Matheus Dias Cordeiro, Claudia Bezerra da Silva, Liani Coronado Báez, Dasiel Obregón, Daniel Moura de Aguiar, Amanda Noeli da Silva Campos, Ísis Indaiara Gonçalves Granjeiro Taques, Alejandra Wu-Chuang, Eugenio Roque López, Elianne Piloto-Sardiñas, Lianet Abuin-Denis, Adivaldo Henrique da Fonseca, Alejandro Cabezas-Cruz

**Affiliations:** 1 Department of Preventive Veterinary Medicine, Agrarian University of Havana, Carretera de Tapaste y Autopista Nacional, Km 23 1/2, 32700 San José de las Lajas, Mayabeque Cuba; 2 Department of Pathobiology, Institute of Parasitology, University of Veterinary Medicine Vienna, Veterinaerplatz 1, 1210 Vienna, Austria; 3 Direction of Animal Health, National Center for Animal and Plant Health, Carretera de Tapaste y Autopista Nacional, Apartado Postal 10, 32700 San José de las Lajas, Mayabeque Cuba; 4 Department of Animal Parasitology, Federal Rural University of Rio de Janeiro (UFRRJ), BR 465, Km 7, Seropedica, RJ 23890000 Brazil; 5 School of Environmental Sciences, University of Guelph, Guelph, ON N1G 2W1 Canada; 6 Virology and Rickettsioses Laboratory, Hospital Veterinário da Facultade de Medicina Veterinária, Federal University of Mato Grosso State, Cuiabá, Mato Grosso Brazil; 7 UMR BIPAR, INRAE, ANSES, Ecole Nationale Vétérinaire d’Alfort, Université Paris-Est, 94700 Maisons-Alfort, France; 8 Animal Biotechnology Department, Center for Genetic Engineering and Biotechnology, Avenue 31 between 158 and 190, P.O. Box 6162, 10600 Havana, Cuba; 9 Department of Epidemiology and Public Health, Federal Rural University of Rio de Janeiro (UFRRJ), BR 465, Km 7, Seropedica, RJ 23890000 Brazil

**Keywords:** Ticks, *Ehrlichia canis*, Canine monocytic ehrlichiosis, Dogs, *trp36*, *Rhipicephalus sanguineus*

## Abstract

**Background:**

Canine monocytic ehrlichiosis (CME) is caused by the tick-borne pathogen *Ehrlichia canis*, an obligate intracellular Gram-negative bacterium of the family Anaplasmataceae with tropism for canine monocytes and macrophages. The *trp36* gene, which encodes for the major immunoreactive protein TRP36 in *E. canis*, has been successfully used to characterize the genetic diversity of this pathogen in different regions of the world. Based on *trp36* sequence analysis, four *E. canis* genogroups, United States (US), Taiwan (TWN), Brazil (BR) and Costa Rica (CR), have been identified. The aim of this study was to characterize the genetic diversity of *E. canis* in Cuba based on the *trp36* gene.

**Methods:**

Whole blood samples (*n* = 8) were collected from dogs found to be infested with the tick vector *Rhipicephalus sanguineus* sensu lato (s.l.) and/or presenting clinical signs and symptoms of CME. Total DNA was extracted from the blood samples and *trp36* fragments were amplified by PCR. Nucleotide and protein sequences were compared using alignments and phylogenetic analysis.

**Results:**

Four of the *trp36* sequences obtained (*n* = 8) fall within the phylogenetic cluster grouping the US genogroup *E. canis* strains. The other *E. canis trp36* sequences formed a separate and well-supported clade (94% bootstrap value) that is phylogenetically distant from the other major groups and thus represents a new genogroup, herein designated as the ‘Cuba (CUB) genogroup’. Notably, dogs infected with the CUB genogroup presented frequent hemorrhagic lesions.

**Conclusions:**

The results of this study suggest that genetic diversification of *E. canis* in Cuba is associated with the emergence of *E. canis* strains with increased virulence.

## Background

*Ehrlichia canis* is an obligate intracellular bacterium transmitted by the brown dog tick, *Rhipicephalus sanguineus* sensu lato (s.l.). The bacterium is the primary etiologic agent of canine monocytic ehrlichiosis (CME), a serious and sometimes lethal tick-transmitted rickettsial disease affecting mainly domestic dogs [1]. The acute disease is characterized by a high fever, depression, lethargy, anorexia, lymphadenomegaly, splenomegaly and hemorrhagic tendencies (usually exhibited by dermal petechiae and ecchymoses, and epistaxis). Ophthalmological lesions are frequent and include anterior uveitis, chorioretinitis, papilledema, retinal hemorrhage, presence of retinal perivascular infiltrates and bullous retinal detachment [2].

Although *E. canis* is primarily associated with canine disease, human infections with this pathogen have been reported, originally in Venezuela [3] and more recently in Panama [4]. In addition, *E. canis* DNA was detected in samples from human blood-bank donors in Costa Rica [5]. Molecular characterization of *E. canis* has been accomplished using highly conserved genes such as the* 16S* ribosomal RNA (rRNA) and disulfide oxidoreductase (*dsb*), as well as other immunoreactive protein gene sequences, including those of the OMP-1 family (p28/30). Despite the wide geographic dispersion of *E. canis*,* 16S* rRNA gene sequences are 99.4–100% identical among isolates from South America, North America, Asia, Europe, Africa and the Middle East. This close similarity between *E. canis 16S* rRNA genes provides little information regarding the overall diversity of this organism. Similarly, the immunoreactive proteins, including those of the OMP-1 family, DSB, TRP19 and TRP140, have also been found to be conserved in geographically dispersed strains [6–11].

The* trp36 *gene, which encodes a major Tandem Repeat Protein (TRP), TRP36, provides more information regarding *E. canis* genetic diversity and can be used for genotyping *E. canis* strains based on amino acid tandem repeat sequences and/or on the numbers of tandem repeats [5, 12, 13]. *Ehrlichia canis* TRP36 contains a major antibody epitope in the tandem repeat region [14], and ehrlichial TRPs are major immunoreactive proteins that have been associated with functional host–pathogen interactions such as adhesion and internalization, actin nucleation and immune evasion [15]. Variations in the sequence and/or number of tandem repeats of TRP36 may alter the biological function of this protein, possibly resulting in different forms of disease presentation [16, 17].

Phylogenetic analysis of *trp36* gene sequences has allowed the distinction of four *E. canis* genogroups: (i) the USA (US) genogroup, identified in North America, Brazil, Nigeria, Cameroon, Spain, Turkey and Israel [11, 13, 16]; (ii) the Taiwan (TWN) genogroup, identified in South Africa, Thailand, Turkey and Taiwan [18, 19]; (iii) the Brazil (BR) genogroup, identified in the midwest, northern and southern regions of Brazil and recently in Turkey [17, 20]; and (iv) the Costa Rica (CR) genogroup, recently detected in human blood donors from Costa Rica [5] and described in canines from four Peruvian settlements [21].

In Cuba, the first published studies on tick-borne diseases of dogs were carried out by Pérez et al. [22] who described a case of CME, based on clinical and pathological findings. León et al. [23] studied 155 dogs, of which 82.5% were seropositive for *E. canis*, and observed rickettsia-like structures in blood smears from 13 of them.

More recently, an epidemiological study including 378 domestic dogs from four municipalities in the western region of Cuba found high prevalence (47.4%) of *E. canis* infection detected by PCR in blood samples [24]. In addition, of 206 plasma samples examined by indirect enzyme-linked immunosorbent assay (ELISA), 78.6% were seropositive for *E. canis* [24]. An increased risk of *E. canis* infection in some localities with a history of tick infestation was also observed [24]. In another study, tick infestation on dogs was assessed in the western region of Cuba, revealing that 40% of dogs were infested by ticks morphologically characterized as *R. sanguineus* s.l. [25]. A subsequent epidemiological study conducted in the same municipalities detected a high prevalence of *E. canis* in dogs, which provided strong evidence that *R. sanguineus* s.l. is the vector of *E. canis* in Cuba [26], as in other regions of the world [27–29]. Phylogenetic analysis based on* 16S* rRNA, and *glt*A genes suggested a low genetic diversity of *E*. *canis* in Cuba [30], but molecular markers with higher genetic resolution, such as the* trp36* gene, may provide a more realistic view of the genetic diversity of this important pathogen in the country. The present study aimed to determine the genetic diversity of *E. canis* in Cuba using *trp36* gene sequences.

## Methods

### Samples

A cross-sectional study was conducted between October and November 2013 to assess the prevalence of the tick-borne pathogen *E. canis* in dogs from four municipalities located in the western region of Cuba [24]. In total, 378 dogs were randomly selected to assess infection status and seroprevalence of *E. canis* in Cuba. Blood samples were collected in dogs regardless of sex, breed, age or presence of clinical symptoms related to CME. The sample size per municipality was 104 dogs in Habana del Este (Province La Habana), 102 dogs in Boyeros (Province La Habana), 82 dogs in Cotorro (Province La Habana) and 90 dogs in San José de las Lajas (Province Mayabeque). Of these 378 dogs, 179 were positive for *E. canis* based on a PCR assay that amplified a region of the* 16S* rRNA [24]. In the present study, blood samples (*n* = 8) collected from dogs confirmed to be positive for *E. canis* infection based on 16S rRNA PCR in the municipalities of Habana del Este (*n* = 3) and Boyeros (*n* = 5) [24] were selected to assess the genetic diversity of *E. canis* in dogs in Cuba. The presence of pathogens other than *E. canis* was not assessed in the samples.

### Clinical diagnostics of canine ehrlichiosis

In accordance with the clinical criteria for the diagnosis of CME established by Navarrete et al. [24], we assessed the following clinical signs for each dog: elevated body temperature, depression, lethargy, anorexia, lymphadenomegaly, splenomegaly and hemorrhagic tendencies (i.e., petechiae and ecchymoses, and epistaxis). We also looked for ophthalmological lesions [2], neurological signs [31], pale mucous membranes and weakness [32].

### Assessment of tick infestation in dogs

The dogs were manually inspected for tick infestation. This assessment was performed primarily to categorize dogs as infested or uninfested. Additionally, a representative sample of any ticks found on a dog, up to 10 specimens per animal, was collected to confirm the tick species on the respective animal. Ticks were deposited into labeled tubes containing 85% ethanol and transported to the laboratory for morphological identification under a dissecting stereoscopic microscope (Carl Zeiss Microscopy GmbH, Jena, Germany) using standard taxonomic keys [33, 34]. Although the collected specimens included immature tick stages, only adult ticks were identified to the species level and developmental stages were not quantified.

### Isolation of *trp36* gene

For the present study, eight* E. canis 16S* rRNA-positive blood samples [24] were tested for *trp36* gene fragment amplification using a heminested PCR. In the first step, the primers TRP36-F2 (forward:5′-TTTAAAACAAAATTAACACACTA-3′) and TRP36-R1 (reverse: 5′-AAGATTAACTTAATACTCAATATTACT-3′) were used to obtain amplicons of 800–1000 base pairs (bp) in a total reaction volume of 25 µl containing 12.5 µl GoTaq®—Green Master Mix 2x (Promega, Madison, WI, USA), 3.0 µl of each primer (10 pmol/µl), 4 µl DNA and 2.5 µl Nuclease Free Water (Promega). The amplification protocol consisted of an initial denaturation at 95 °C for 5 min, 35 cycles of denaturation (95 °C 30 s), annealing (52 °C 30 s) and extension (72 °C for 1 min) and a final extension of 72 °C for 5 min [17]. In the second step, primers TRP36-DF (forward: 5′-CACACTAAAATGTATAATAAAGC-3′) and TRP36-R1 were used [35] under the same conditions as in the first step, except that an annealing temperature of 57 °C applied for 30 s was used. *Ehrlichia canis* strain Cuiaba #1 (kindly donated by the Laboratory of Parasitic Diseases of the Federal Rural University of Rio de Janeiro) was used as the positive control, and ultrapure water was used as the no-template control.

### Amplicon purification and sequencing

The amplicons were subjected to 1.5% agarose gel electrophoresis, stained with GelRed® 10,000X, a red fluorescent DNA gel stain at a concentration of 10,000× in solution (Biotium, Fremont, CA, USA), and examined under ultraviolet (UV) light using a UV transilluminator. The amplified products were purified using the commercial ReliaPrep® DNA Clean-up and Concentration System® Kit (Promega) and sequenced in both directions using the Big Dye Kit™ (Applied Biosystems, Thermo Fisher Scientific, Waltham, MA, USA) by Sanger`s method, according to the manufacturer’s recommendations. The sequences were determined using an automated DNA sequencer ABI 3500 Series Genetic Analyzer (Applied Biosystems), following the instructions of the user manual. The detected sequences were submitted to GenBank.

### Analysis of the *trp36* gene and putative amino acid sequences

The TRP36 protein sequence was evaluated for potential mucin-type* O*-linked glycosylation on serines and threonines with the computational algorithm NetOGlyc v3.1 [36]; for* N*-linked glycosylation, we used the NetNGlyc 1.0 Server (NetNGlyc 1.0 Server, http://www.cbs.dtu.dk/services/NetNGlyc/). The Tandem Repeats Finder (TRF) database [37] was used to predict the presence of tandem repeats in *trp36*. For sequence analysis and comparison, the *trp36* nucleotide and predicted amino acid sequences were divided into three regions (I, II and III) as previously reported [19]. Region I was the 5′-end pre-tandem repeat region composed of 426–429 bp/142–143 amino acids at the N-terminus of the encoded protein; region II was the tandem repeat region (variable numbers of the 27 bp/9 amino acids repeat units depending on the strain); and region III was the 3′-end post-repeat region (81–93 bp/28–30 amino acids) at the C terminus of the encoded protein.

### Phylogenetic analysis

To investigate the phylogenetic relationships among *E. canis trp36* isolates, the representative nucleotide sequences of *E. canis trp36* obtained in this study were compared to those available in GenBank. Multiple sequence alignment was performed using the ClustalW algorithm implemented in the BioEdit software v.7.2.5 [38]. The sequences were trimmed manually, and the resulting overall alignment was 579 bp in length. A neighbor-joining (NJ) tree was constructed applying the Tamura 3-parameter (T92) model [according to the Akaike information criterion corrected for small sample sizes (AICc)] using the MEGA v.7.0 bioinformatics software [39]. Reliability of internal branches was assessed using the bootstrapping method with 1000 bootstrap replicates.

## Results

### Clinical findings associated with canine ehrlichiosis

Five of the dogs tested displayed common clinical signs related with CME (dog5, dog17, dog23, dog78, dog172), including hemorrhagic tendencies, such as petechiae, ecchymoses and epistaxis (dog17, dog23, dog172), cough (dog5) and emaciation (dog78). The three remaining dogs (dog26, dog60, dog92) were asymptomatic. Two of the symptomatic dogs (dog5 and dog23) and two of the asymptomatic dogs (dog60 and dog92) were infested with *R. sanguineus* s.l. (Table 1).

**Table 1:** Clinical findings and tick infestation of dogs

Common clinical signs of canine ehrlichiosis	Clinical signs of canine ehrlichiosis observed in study dogs^a^							
	Dog5^b^	Dog17	Dog23^b^	Dog26	Dog60^b^	Dog78	Dog92	Dog172^b^
Elevation of body temperature	–	–	–	–	–	–	–	–
Depression, lethargy anorexia	–	–	–	–	–	–	–	–
Lymphadenomegaly	–	–	–	–	–	–	–	–
Splenomegaly	–	–	–	–	–	–	–	–
Hemorrhagic tendencies (petechiae and ecchymoses, and epistaxis)	–	X	X	–	–	–	–	X
Ophthalmological lesions	–	–	–	–	–	–	–	–
Neurological signs	–	–	–	–	–	–	–	–
Pale mucous membranes and weakness	–	–	–	–	–	–	–	–
Others clinical signs	Cough	–	–	–	–	Emaciation	–	–
Tick infestation (*Rhipicephalus sanguineus* sensu lato)	X	–	X	–	X	–	X	–

^a^‘X’ indicates the presence of the clinical sign in the dog, and ‘–’ indicates the absence of the clinical sign

^b^Dogs infected with the new CUB genogroup

### Amplification and phylogenetic analysis of* trp36* variants

**Fig. 1:**
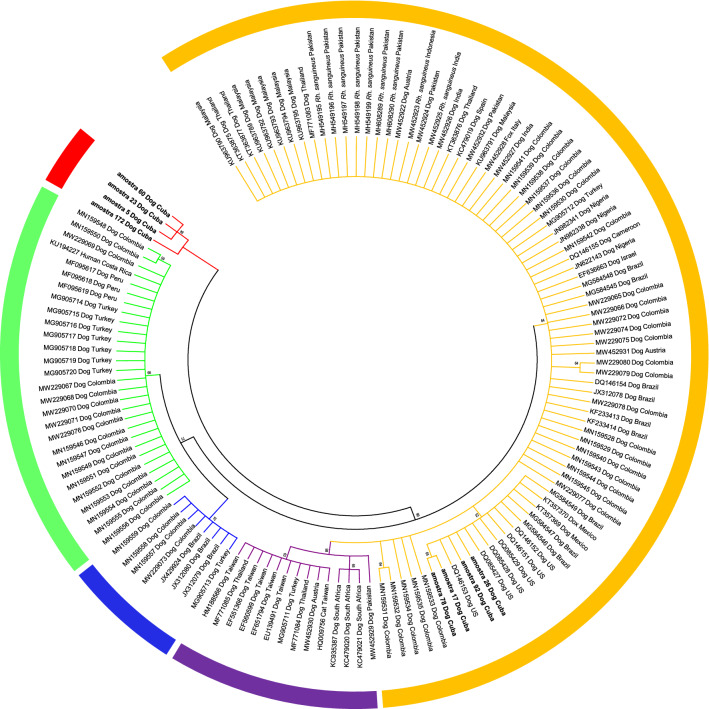
Neighbor-joining tree constructed with the partial *trp36* nucleotide sequences of *E. canis*. Bootstrap values based on 1000 replicates are indicated at the nodes. Only bootstrap values > 50% are included. Sequences generated in this study are indicated in bold. The US, TWN, BR, CR and CUB genogroups are highlighted in orange, violet, blue, green and red, respectively

Partial *trp36* gene sequences were amplified and sequenced from eight *E. canis*-positive blood samples. A NJ phylogenetic analysis using *trp36* nucleotide sequences obtained in this study (*n* = 8) and additional sequences retrieved from GenBank (*n* = 128) showed differential clustering of the sequences into five major clades (Fig. 1). Four of these clades were previously described as the US, TWN, BR and CR genogroups. Notably, four sequences (dog5, dog23, dog60, dog172) formed a separate and well-supported clade (94% bootstrap value) that is phylogenetically distant from the other *E. canis* strains; this clade therefore represents a new genogroup, designated here as the ‘Cuba (CUB) genogroup’. The other four *E. canis trp36* sequences (dog17, dog26, dog78, and dog92) clustered together with sequences of the US genogroup.

### *trp36* sequence analysis

The PCR products derived from the amplified *trp36* gene fragments in the blood samples collected from the different dogs had variable molecular sizes: 465 bp (dog5), 496 bp (dog17), 480 bp (dog23), 516 bp (dog26), 483 bp (dog60), 579 bp (dog78), 479 bp (dog92) and 473 (dog172), encoding for protein sequences of 136, 164, 138, 172, 139, 192, 159 and 149 amino acids, respectively. These predicted amino acid sequences showed variable degrees of identity between them. For example, comparison of some of the sequences showed identity values ranging from 37.41% (dog60 and dog92) to 99.39% (dog17 and dog78).

Comparison of the sequences obtained in this study with previously reported *trp36* sequences showed that samples from dog17, dog26, dog78 and dog92 had an identity between 98.58% and 99.79% compared to other isolates of the US genogroup. To facilitate the molecular analysis, we divided analysis of the TRP36 protein into three regions designated I, II and III. The putative protein sequences of one *trp36* amplicon (dog172) included the three regions. The *trp36* fragments amplified from the other samples only included regions I and II (dog17, dog26, dog78 and dog92) and regions II and III (dog5, dog23 and dog60).

#### TRP36 region I

Comparison of TRP36 fragments in which region I was identified (dog17, dog26, dog78, dog92, dog172) revealed 100% identity between samples from dog17 and dog78 and 100% identity between samples from dog26, dog92 and dog172. The amino acid sequences of region I in samples dog17, dog78 shared 98.46% identity with that in samples dog26, dog92 and dog172.

TRP36 is a glycoprotein [14] containing predicted* N*-glycosylation (region I) and* O*-glycosylation (regions I and II) sites [12, 13]. The addition of* N*-glycosyl groups on asparagine (N) residues requires special motifs called sequons [NX serine (S)/threonine (T)], where X can be any amino acid [40]. The N 125 of the five sequences contained a potential sequon (NPS, where P is proline), but the presence of P between N and S dramatically reduces the probability of* N*-glycosylation [40] and thus it may prevent the addition of glycosyl groups on N 125. In addition, this region presents three S residues in the five samples (dog17, dog26, dog78, dog92 and dog172), which based on prediction are sites of* O*-glycosylation.

#### TRP36 regions II and III

Region II contains a variable number of repeated units of 27 nucleotides coding for nine amino acids depending on the isolate, as reported previously by Doyle et al. [14] and Hsieh et al. [19]. A variable number of tandem repeats (range: 5–12), with a conserved sequence (TEDSVSAPA), was identified in region II of the Cuban isolates (Table 2). Both the nucleotide and amino acid sequences in the tandem repeat region were highly conserved within the Cuban isolates as well as between genogroups (Table 2). The strains reported in this work presented a 100% of amino acid identity in this region when compared to other isolates (Table 2). The samples from dog23 and dog60 have no defined last amino acid in four and two tandem sequences, respectively, but the nine amino acid consensus sequences rarely present amino acid changes [9]. Region II is rich in S and T residues, which could be potential* O*-glycosylation sites. The length of region III was 30 amino acids for samples from dog5, dog23, dog60 and dog172, with 100% of identity between them and in comparison with other samples.

**Table 2:** Summary of *trp36* sequence features from representative strains of different *Ehrlichia canis* genogroups

Genogroups	Representative strains (accession number)	Tandem repeats						
		Nucleotide sequence			Protein sequence			Amino acid sequence
		Length (bp)	No.^b^	Identity (%)^c^	Length (amino acids)	No.^b^	Identity (%)^c^	
Brazil	MG584546	27	11	100	9	11	100	TEDSVSAPA
Taiwan	EU139491	27	14	100	9	14	100	TEDSVSAPA
US	DQ146153	27	18	100	9	18	100	TEDSVSAPA
Cuba	Dog5^a^ (ON231837)	27	12	100	9	12	100	TEDSVSAPA
	Dog17^a^ (ON231838)	27	7	100	9	7	100	TEDSVSAPA
	Dog23^a^ (ON231839)	27	12	100	9	12	100	TEDSVSAPA
	Dog26^a^ (ON231840)	27	7	100	9	7	100	TEDSVSAPA
	Dog60^a^ (ON231841)	27	12	100	9	12	100	TEDSVSAPA
	Dog78^a^ (ON231842)	27	8	100	9	8	100	TEDSVSAPA
	Dog92^a^ (ON231843)	27	5	100	9	5	100	TEDSVSAPA
	Dog172 (ON231844)	27	6	100	9	6	100	TEDSVSAPA

^a^Partial sequence

^b^Number of tandem repeats

^c^Percentage of tandem repeats identity within a sequence

## Discussion

The intracellular bacteria *Ehrlichia canis* is globally distributed and is the most common tick-borne pathogen infecting dogs in South America [16, 41–43]. Infection with *E. canis* has been reported previously in Cuba [22], and phylogenetic analysis based on *E. canis 16S* rRNA led to the identification of 179 *E. canis*-positive dogs in the western region of Cuba [24]. In the present study, a fragment of *trp36* was amplified from eight *E. canis*-positive blood samples collected from dogs and sequenced to characterize the genetic diversity of this bacterium in Cuba.

The *trp36* gene has significant diversity and allows the differentiation of *E. canis* genotypes isolated in different geographic locations [19, 44]. Several *E. canis* strains of the US, CR and BR genogroups have been reported in South America. For example, strains of the US genogroup have been reported in Brazil and Venezuela; while other strains within the CR and BR genogroups were reported in Peru and Costa Rica [5, 21] and in Brazil [16], respectively. Notably, four strains identified in this study formed a clade separated from all currently known genogroups, revealing the presence of strains from two *E. canis* genogroups in Cuba, the US genogroup and the CUB genogroup, reported here for the first time. A more exhaustive sampling may have revealed the presence of additional genogroups in the country. For example, *trp36* gene sequencing in 35 samples of *E. canis*-positive dogs revealed the presence of three genogroups (i.e., US, CR, and BR) in Colombia [45]. The evolutionary events associated with the emergence of the CUB genogroup are not clear and are beyond the scope of this study. However, genetic diversification of *E. canis trp36* has been linked with episodic bursts of selection unequally distributed across nucleotide positions [5]. The *trp36* gene was under strong selection in highly diverse *E. canis* strains [5] identified in South Africa [12] and Brazil [17]. We propose that episodic diversifying selection, such as that affecting highly diverse *E. canis* strains in South Africa and Brazil, may have contributed to the diversification of *E. canis* in Cuba.

The presence in Cuba of *E. canis* strains of the US genogroup, the most frequent among canids and tick vectors [46], suggests pathogen introduction events, probably associated with host movement (e.g., infected dogs) and/or tick vector migration (i.e., infected ticks carried by migratory birds). Dogs with chronic, subclinical *E. canis* infection can be transported to new locations and serve as reservoirs for pathogen acquisition by local *R. sanguineus* s.l. ticks. Records show that traveling dogs are not fully protected and/or free of infection since cases of vector-borne diseases, including CME caused by *E. canis*, occur in non-endemic regions [47]. Despite parasitism by *R. sanguineus* s.l. on hosts other than dogs is unusual, this tick can occasionally infest a wide range of domestic and wild hosts, including cats, rodents and birds, as well as humans [48]. Thus, *R. sanguineus* s.l. ticks infected with *E. canis* and/or *E. canis*-infected hosts (e.g., dogs or migratory birds) could have been associated with the presence of the US genogroup strains in the country.

In agreement with other studies [12, 17, 45, 46], our results support the use of the *trp36* gene as a suitable molecular tool for genotyping *E. canis*, based not only on phylogenetic analysis of *trp36* nucleotide sequences, but also on differences in the amino acid sequences of regions I, II and III, as well as on the number and sequence of the TRP36 tandem repeats. Analysis of TRP36 region I showed the presence of N 125 in the context of a potentially non-glycosylated sequon, NPS, previously identified in *E. canis* strains in the USA, Spain, Israel, Central Africa and Brazil [5]. In contrast, in strains from Taiwan and South Africa, N 125 is present in the context of a potentially glycosylated sequon, NSS [5]. The relevance of sequon sequence variability and of the eventual absence (NPS) or presence (NSS) of glycosylation for *E. canis* life cycle and/or pathogenicity are currently unknown. However, as* N*-glycosylation plays an important role in cellular biology, impacting on several properties of proteins, such as solubility, stability and turnover, secretion, protease resistance, protein–protein interaction/recognition and immunogenicity [40], differences in glycosylation patterns contribute to evasion of the host immune system [13, 49] and antigenic drift [5, 50]. Whether variations in TRP36 glycosylation overlap differences in *E. canis* pathogenicity warrants further investigations.

## Conclusions

Taken together, the results of this study provide important information on the genetic diversity of *E. canis* in Cuba, reporting for the first time the characterization of *trp36* gene fragments of *E. canis* strains identified in the country as well as the presence of a new *E. canis* genogroup, named the CUB genogroup. The combination of clinical findings and genetic diversity analysis revealed that animals infected with strains of the CUB genogroup presented hemorrhagic tendencies (dog23 and dog172) and cough (dog5). This suggests that *E. canis* strains of the CUB genogroup could be associated with increased virulence and pathogenicity in dogs with CME in Cuba, a hypothesis that warrants further research.

## Data Availability

The nucleotide sequences obtained in this study were submitted to GenBank and are available under the accession numbers ON231837 (dog5), ON231838 (dog17), ON231839 (dog23), ON231840 (dog26), ON231841 (dog60), ON231842 (dog78), ON231843 (dog92), and ON231844 (dog172).
